# P-832. The Skip Phenomenon in Patients with *Staphylococcus lugdunensis* Bloodstream Infection

**DOI:** 10.1093/ofid/ofae631.1024

**Published:** 2025-01-29

**Authors:** Luis R Gasca, Patrick Crowley, Nicholas Streck, Larry M Baddour, Daniel C DeSimone

**Affiliations:** Mayo Clinic, Rochester, Minnesota; Mayo Clinic, Rochester, Minnesota; Mayo Clinic, Rochester, Minnesota; Mayo Clinic College of Medicine, Rochester, MN; Mayo Clinic, Rochester, Minnesota

## Abstract

**Background:**

*Staphylococcus lugdunensis* (*SL*) is a coagulase-negative staphylococcus with virulence comparable to that of *Staphylococcus aureus*. The “skip phenomenon” (SP) is a pattern where blood cultures are intermittently negative before final clearance of bloodstream infection (BSI). SP is well-documented in *S. aureus* bacteremia but has not been described previously in *SL*. In this report, we present a cohort with *SL* BSI and describe its prevalence and their clinical features and outcomes.

**Methods:**

We conducted a retrospective review of patients with blood cultures positive for *SL* in samples referred to the Mayo Clinic reference lab between June 2012 and June 2022. We assessed clinical characteristics, including time to positivity, prevalence of the skip phenomenon (defined by a positive culture after 24 hours of negative cultures), patient demographics including Charlson Comorbidity Index, mortality and length of hospital stay.

**Results:**

We identified 55 patients with *SL* BSI and 33 of them had enough laboratory data to determine the prevalence of the skip phenomenon. SP was present in 5 of 33 (15.2%) patients. For all patients with *SL* BSI, the median age was 67 years (interquartile range [IQR] 61-79); mean CCI in the group with SP was 5.6 (standard deviation 2) and those without SP was 5.9 (standard deviation 2.7); median duration of bacteremia was 3 days (IQR 1-4); median hospital length of stay was 14 days (IQR 8-20); and 1-year mortality was 45%. Oxacillin susceptibility was present in 33 of 34 cases.

**Conclusion:**

A total of 15% of patient cases with *SL* BSI demonstrated the SP. Recognition of the SP in patients with *SL* BSI is important as this may impact duration of antibiotic therapy and timing of long-term cardiovascular device placement.

**Disclosures:**

**Larry M. Baddour, MD**, UpToDate, Inc.: Royalty payments (authorship duties).

